# A Δ11 desaturase gene genealogy reveals two divergent allelic classes within the European corn borer (*Ostrinia nubilalis*)

**DOI:** 10.1186/1471-2148-10-112

**Published:** 2010-04-27

**Authors:** Kerry A Geiler, Richard G Harrison

**Affiliations:** 1 Department of Organismic and Evolutionary Biology, Harvard University, Biological Laboratories, Divinity Road, Cambridge, MA USA; 2 Department of Ecology and Evolutionary Biology, Cornell University, Corson Hall, Ithaca, NY USA

## Abstract

**Background:**

Moth pheromone mating systems have been characterized at the molecular level, allowing evolutionary biologists to study how changes in protein sequence or gene expression affect pheromone phenotype, patterns of mating, and ultimately, the formation of barriers to gene exchange. Recent studies of *Ostrinia *pheromones have focused on the diversity of sex pheromone desaturases and their role in the specificity of pheromone production. Here we produce a Δ11 desaturase genealogy within *Ostrinia nubilalis*. We ask what has been the history of this gene, and whether this history suggests that changes in Δ11 desaturase have been involved in the divergence of the E and Z *O. nubilalis *pheromone strains.

**Results:**

The Δ11 desaturase gene genealogy does not differentiate *O. nubilalis* pheromone strains. However, we find two distinct clades, separated by 2.9% sequence divergence, that do not sort with pheromone strain, geographic origin, or emergence time. We demonstrate that these clades do not represent gene duplicates, but rather allelic variation at a single gene locus.

**Conclusions:**

Analyses of patterns of variation at the Δ11 desaturase gene in ECB suggest that this enzyme does not contribute to reproductive isolation between pheromone strains (E and Z). However, our genealogy reveals two deeply divergent allelic classes. Standing variation at loci that contribute to mate choice phenotypes may permit novel pheromone mating systems to arise in the presence of strong stabilizing selection.

## Background

The origin of novel sex pheromone signaling systems may play an important role in insect speciation. Insect sex pheromones are volatile compounds or mixtures of such compounds, used in many species for mate location, species recognition, and mate choice [1]. In many moths, females produce species-specific chemical cues, and males exhibit species-specific responses (both physiological and behavioral) that are important in mate finding. Males may also produce pheromones that are used by females in exercising mate choice (e.g., [2]). These chemical cues are often blends of long-chain hydrocarbons with acetate, alcohol, or aldehyde functional groups. Because pheromone biosynthetic pathways have been well characterized [3,4], it is now possible to examine how changes at the level of protein sequence or gene expression affect pheromone phenotype, patterns of mating, and ultimately, the nature and origin of barriers to gene exchange.

Pheromone signaling systems are described as "highly canalized" because changes in production or response are opposed by strong selection against novel phenotypes [5,6]. Only if the same genes control signal and response (pleiotropy), or if the genes controlling these traits are tightly linked, is coordinated evolution of signal and response thought to be likely. Given the diversity of pheromone blends (and responses) within closely related groups, it is clear that novel signaling systems arise regularly; however, in moth mating systems studied to date, signal and response genes appear to be unlinked [7]. Several models have been proposed to explain how novel sex pheromones could evolve without linkage or pleiotropy. In these models, pre-existing variation in either pheromone production or response increases the likelihood that novel pheromone blends or preferences will result in successful mating [5,6].

Moths in the genus *Ostrinia* (Crambidae) have provided an important model system for examining the evolution of pheromone communication [8-10]. In this genus, female pheromone blends are known for many of the species, the biosynthesis of these components is well understood, and differences in pheromone blends between species or strains are consistent with determination by single major genetic factors [5,6,10-13]. The principal components of *Ostrinia* female pheromones are unsaturated 14-carbon acetates ((Z/E)-11-tetradecenyl or (Z/E)-12-tetradecenyl acetate), which are synthesized from 16C-acids through a series of enzymatic steps that result in chain shortening, desaturation, reduction, and acetylation [3,4,14]. Not only do species differ in pheromone blend, but within the well-studied European corn borer (ECB), two distinct "pheromone strains" (E and Z) occur [15]. In the E strain, females produce and males respond to a 99:1 mixture of the E and Z isomers of D11-14:OAc; in the Z strain the proportions are reversed and females produce and males respond to a 3:97 mixture [16]. Pheromone blends produced by male hairpencils also differ between strains and species in *Ostrinia*[2]. Although differentiated with respect to pheromone communication, the E and Z strains are otherwise difficult to distinguish, using either morphological traits [17,18] or molecular characters [19-22]. Most gene genealogies for ECB do not reveal the two strains to be exclusive groups; only at one locus (Tpi, triose phosphate isomerase) and surrounding chromosomal regions is there substantial differentiation [[22,23], unpublished data].

Recent studies of *Ostrinia* pheromones have focused on the diversity of sex pheromone desaturases and their role in the specificity of pheromone production [2,6,10,13,24-26]. In the European and Asian corn borers (*Ostrinia nubilalis* and *O. furnacalis*) the pheromone desaturases, together with desaturases involved in fatty acid metabolism, comprise a large multi-gene family. Distinct subfamilies exist that differ in specificity and localization (e.g., fat body versus pheromone gland). In ECB, the Δ11 desaturase subfamily includes at least 10 members, about half of which are full-length copies; the remainder appear to be truncated and lack exon 1 [25].

Here we produce a Δ11 desaturase gene genealogy within ECB, based on sequence from the two putative functional genes found in Xue et al. [25]. Despite the presence of multiple Δ11 desaturase gene copies, previous studies of E and Z pheromone gland extract suggest that the pheromone strains do not differ in Δ11 desaturase enzyme activity [27,28]. We use genealogical methods to confirm whether or not the E and Z strains are differentiated at the Δ11 desaturase locus. We also examine whether the two functional Δ11 desaturase candidates represent gene duplicates or allelic variation at a single locus. Finally, we use population genetic methods to ask what has been the history of the gene(s) in ECB. We interpret our results in light of ECB demography and the recent demonstration of a dual function of Δ11 desaturase in both male and female pheromone biosynthesis [2].

## Results

### Gene structure and polymorphism

**Figure 1:**
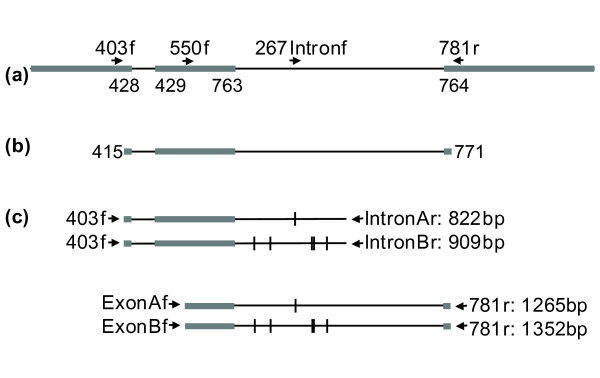
**Structure of the ECB Δ11 desaturase gene, primer locations, and regions amplified**. **(a) **The complete Δ11 desaturase gene, showing primer locations. Exons are shown as gray bars, introns as black lines, primers as arrows. Numbers below the intron/exon boundaries indicate positions of intron start and end points in the Δ11 desaturase coding sequence. **(b) **Region sequenced for genealogical analysis. The region between base pairs 415-771 of the coding region contains 357 bp of exon (11 bp exon 1, 338 bp exon 2, 8 bp exon 3) and 1008-1988 bp of intron (105-279 bp intron 1, 903-1883 bp intron 2). **(c) **Size of expected products from clade specific PCR. Four PCR products of different lengths are shown. Each results from a PCR reaction using the specified primer pairs. For each primer pair, one primer matches sequence only from clade A or clade B. Locations of the six indels responsible for the 87 bp length difference between clades are indicated with vertical dashes.

We sequenced 1365 bps of the Δ11 desaturase gene from cDNA bps 415-771 in 38 corn borers (36 ECB, 2 ACB). Amplification and sequencing from genomic DNA, using primers shown in Table 1, confirms the presence of two introns. The first, at base pair 428, ranges from 105 to 279 bps in length, while the second, at base pair 763, ranges from 903 to 1883 bps in length (Figure 1). Intron length varies due to the presence of multiple indels, some of which are large (Additional File 1: LargeInsertions.pdf). Figure 1 displays the intron length when all indels are coded as a single base. Numbering of intron start positions is based on the entire mRNA sequence, not coding DNA.

**Table 1:** Primers used for sequencing and for determining in which clade alleles fall.

Primer name	Purpose	Sequence
403f	amplification and sequencing	GCTGTATTTGGGATTTACATCAG

781r	amplification and sequencing	TGGAACGCTTTGTTTCGTTCTC

550f	sequencing	CATRGTGTTTAACAGCATGGC

I267f	sequencing	AATACCCTCWGCAAAAGTCTA

ExonAf	A clade specific	**T**TATGGAG**C**CA**C**AGAAGCTA**C**

ExonBf	B clade specific	**C**TATGGAG**T**CA**T**AGAAGCTA**T**

IntronAr	A clade specific	G**T**CGGTC**ATTGG**GTG**T**TTGTAG

IntronBr	B clade specific	G**C**CGGTC**TAGTT**GTG**C**TTGTAG

Mismatches between clade specific primers are in bold.

The amount of polymorphism within our ECB sample set is similar to that found at other ECB loci [21,22]. We find 161 polymorphic sites within the 36 ECB sequences, 41 indel polymorphisms and 120 single base substitutions (Table 2).

**Table 2:** Analyses of polymorphism with tests of selection.

	Variable sites (S)	Parsimony informative sites	Nucleotide diversity π	Tajima's D	Fu and Li's D	Fu and Li's F	Net divergence between populations D_a_
All ECB	120	59	0.017 (0.0010)	-0.80	-2.77 **P < 0.02**	-2.43 **P < 0.05**	

E moths	74	38	0.019 (0.0015)	0.11	-0.965	-0.078	E and Z: 0.00015

Z moths	94	49	0.017 (0.0016)	-0.52	-1.906	-1.703	

Clade A	55	17	0.007 (0.0005)	-1.53	ACB1: -3.218 **P < 0.02** ACB2: -2.700 P < 0.02	ACB1: -3.230 **P < 0.02** ACB2: -2.800 P < 0.05	A and B: 0.023

Clade B	33	8	0.005 (0.0008)	-1.60	ACB1: -2.748 **P < 0.02** ACB2: -2.135 0.10 > P > 0.05	ACB1: -2.952 **P < 0.02** ACB2: -2.413 P < 0.05	

Analyses omit sequence positions containing a gap and do not include insertion deletion polymorphism. Standard deviation of nucleotide diversity is in parentheses. Fu and Li's D and F statistics (Fu and Li 1993) were calculated using the total number of segregating sites. If choice of ACB1 vs. ACB2 as an outgroup gave different values, both are reported. P values are given for test statistics that deviate from neutral expectations.

### Analysis of gene genealogies

**Figure 2:**
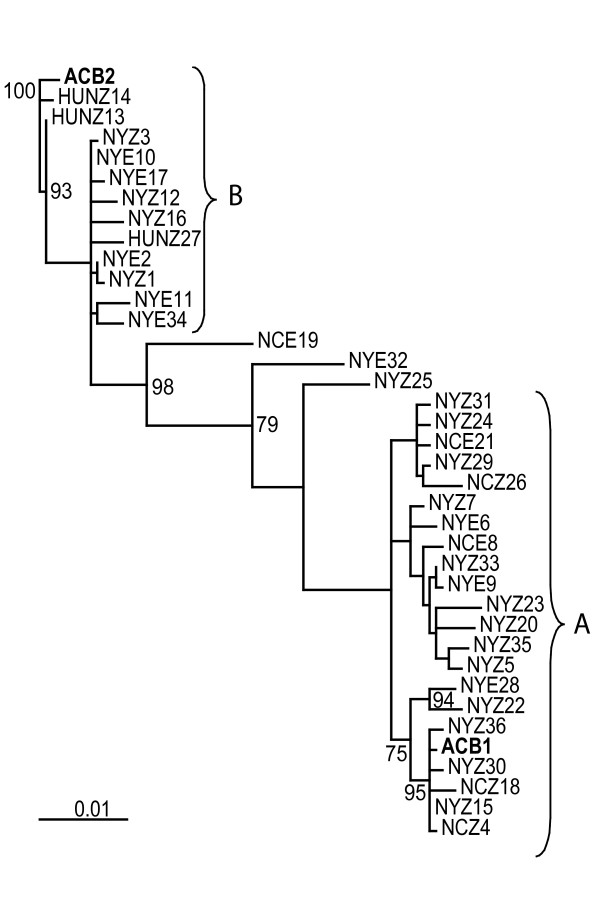
**Δ11 desaturase gene genealogy for ECB, using ACB as an 'outgroup'**. Shown is the maximum parsimony gene genealogy with ACB sequences labeled in bold and major clades denoted "A" and "B". ECB individuals are labeled with abbreviations for states or countries (NY = New York, NC = North Carolina, HUN = Hungary) and with Z or E to indicate to which pheromone strain they belong. ACB2 was chosen as an outgroup (as opposed to ACB1) because ACB2 is similar to ECB alleles from the European population in Hungary. Bootstrap values are indicated based on analysis using 1,000 replicates. Values less than 75% are not shown.

The Δ11 desaturase genealogy does not reveal the two ECB pheromone strains (E and Z) to be exclusive (monophyletic) groups (Figure 2). There are no fixed nucleotide or fixed indel differences between pheromone strains. The estimated average pairwise distance between E and Z populations (Da in [29]) is 0.00015 (Table 2).

The Δ11 desaturase maximum parsimony genealogy documents the presence of two major clades (A and B) that differ by six insertion/deletions, resulting in an 87 bp length difference. These clades do not separate E and Z borers, nor do they sort moths based on geographic origins or life cycles (univoltine versus bivoltine). Trees based on only intron or only exon sequences show the same deep divergence between the two clades (Additional File 2: ExonIntronTree.pdf), as do regional trees based on 5', middle and 3' regions of the desaturase gene (Additional File 3: RegionalTrees.pdf) and the maximum likelihood genealogy (Additional File 4: MaximumLiklihoodTree.pdf). Bootstrap analysis demonstrates that 98.4% of genealogies include the branch separating the two clades (Figure 2).

The major clades (A and B) in the Δ11 desaturase genealogy correspond to the two full length Δ11 desaturase duplicates (Short "S" and Long "L") discovered by Xue et al [25]. Alignments of intron 2 from clades A and B and from haplotypes S and L show the same pattern of six insertion/deletions, with clade B and haplotype L containing 87 additional nucleotides. We find that most (59) of the 73 nucleotide sites in intron 2 reported as fixed differences between the S and L sequences by Xue et al. [25] are segregating within clades A or B. This discrepancy is likely the result of the increased sample size of moths in our data set. Xue et al. [25] found that exon 2 was identical in the S and L haplotypes. In contrast, we find that exon sequences also differ between clades A and B. Of 24 segregating sites in exon 2, 10 are fixed nucleotide differences between clades. At all 10 of these sites, our clade A exon matches the published ECB Δ11 desaturase sequence in Genbank (AF441221) [6]. The exon 2 sequences from clade B match two published ECB Δ11 desaturase sequences from French populations (EU350083-EU350084) [2].

### Evidence for gene duplication?

We tested whether clades A and B represent gene duplicates or allelic variation at a single locus using clade specific PCR. We find that some moths have both A and B haplotypes (where A and B refer to clade membership), whereas other moths appear to have haplotypes that fall into only one clade (either A or B) (Additional File 5: CladeSpecificResults.xls). Thus, our data fail to show the fixed heterozygote pattern expected for duplicate genes (every moth would possess copies of both A and B haplotypes). If we assume that the variation we see is allelic variation at a single locus, then the inferred numbers of genotypes are 5 moths homozygous for B, 10 homozygous for A, and 12 heterozygotes (AB). These genotype frequencies do not include 9 of the 36 moths. These 9 moths were shown, by sequencing (3 moths) or clade specific PCR (6 moths), to carry at least one recombinant haplotype.

### Evidence for recombination

**Figure 3:**
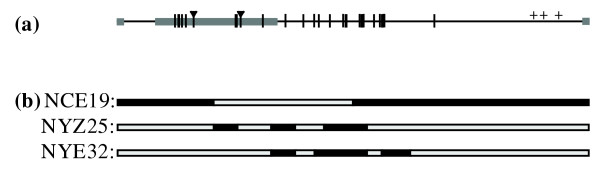
**Differences between clades and estimated recombination breakpoints**. **(a**) Fixed differences between clades A and B. The region shown is from bp 415 to bp 771, with exons represented as gray bars and introns as horizontal black lines. Vertical dashes represent fixed differences between the A and B clades. Triangles above exon positions 224 and 363 mark the two amino acid substitutions. "+" symbols indicate three polymorphisms that would be fixed differences except for one allele in NCZ26. **(b) **Recombination breakpoints for sequences NCE19, NYZ25, NYE32. These inferred recombinant alleles share similarity to both clades A and B for different parts of the Δ11 desaturase gene region. Light bars indicate identity to clade A, and dark bars indicate identity to clade B. Recombination breakpoints are estimated from the fixed differences between clades A and B and are displayed as the midpoint between two fixed differences for which the recombinant's haplotype changes from one clade to the other. Most stretches of similarity are supported by multiple fixed differences (compare to (a) to see how many sites (vertical dashes) support that stretch of similarity). Two inferred recombination events are supported by only one nucleotide change and may represent point mutations.

Three ECB moths (NCE19, NYZ25, and NYE32) fail to sort with either of the two major clades (Figure 2), and (with the exception of NCZ26 in the 3' gene tree) are the only haplotypes for which position in the genealogy changes substantially in the three regional Δ11 desaturase genealogies (Additional File 3: RegionalTrees.pdf). Alignment of these sequences suggests that they are recombinant haplotypes, (i.e., they possess long stretches of similarity to sequences from clade A, followed by a long region of similarity to sequences from clade B) (Figure 3). Repeated amplification and sequencing of these three moths confirms that this is not PCR induced recombination. Six additional moths were shown to carry at least one recombinant haplotype by clade specific PCR (Additional File 5: CladeSpecificResults.xls). For the complete panel of 36 moths (72 haplotypes), 24 haplotypes are from clade B, 37 haplotypes are from clade A, with 11 presumed recombinant haplotypes. To quantify the amount of recombination indicated by our genealogy, we estimated the minimum number of recombination events (Rm) to be 8 [30] and the number of crossover events to be 8.6. We also use C [31] and γ [32] to estimate recombination per base pair, and find these values (C = 0.006; γ = 0.0078) to be lower than those estimated for a different ECB sample at the gene encoding pheromone binding protein (C = 0.031; γ = 0.017) [21].

### Polymorphism and genetic distance between clades

Despite evidence that clades A and B represent alleles at the same locus, and direct evidence of recombination at this locus, clades A and B retain many fixed differences and little shared polymorphism (Table 3). Only 3 of 109 single nucleotide polymorphisms (SNPs) are polymorphic in both clades A and B. Conversely, 82 of these SNPs segregate within one clade but are not polymorphic in the second clade (52 within clade A and 30 within clade B). The final 24 single base substitutions represent fixed differences between the two major clades: 14 single nucleotide substitutions in intron 2 and 10 single nucleotide substitutions in exon 2 (Figure 3). Of the 10 fixed differences in the exon, 2 represent fixed amino acid differences: M102I and M149L. These represent conservative changes, inter-conversions among the nonpolar, neutral residues methionine, isoleucine, and leucine. The total number of fixed differences between clades A and B is 30 (24 SNPs and 6 indels). Three additional SNPs (+'s in Figure 3) would be fixed differences if not for NCZ26. These may be evidence of a recombination event at the 3' end of NCZ26.

**Table 3:** Numbers of single nucleotide polymorphisms within and between clades A and B.

	Total sites	Polymorphic Sites	Fixed Differences	Polymorphic A Monomorphic B	Polymorphic B Monomorphic A	Shared Polymorphism
3' Exon 1	11	1	0	1	0	0

Intron 1	105	11	0	8	3	0

Exon 2	338	24	10 (2R/8S)	11 (5R/6S)	2 (1R/1S)	1

Intron 2	903	73	14	32	25	2

5' Exon 3	8	0	0	0	0	0

**Total**	**1365**	**109**	**24**	**52**	**30**	**3**

These counts do not include the three recombinant sequences; therefore, the total number of polymorphic sites is less than is shown in Table 2 (recombinant sequences add 11 polymorphic sites). Also, this analysis omits any sequence position containing a gap and therefore does not include insertion deletion polymorphism. R = replacement substitution and S = silent substitution.

The desaturase gene genealogy is the fifth nuclear gene genealogy for ECB constructed using an almost identical panel of 36 moths [22]. Nucleotide diversity in this genealogy (π = 0.017) is within the range defined by previous estimates at the other four loci (Kettin, π = 0.006, Ldh, π = 0.026, Pbp, π = 0.021, Tpi, π = 0.004). The nucleotide diversity within either clade A or B is at the low end of this spectrum (π_A _= 0.007, π_B _= 0.005), whereas average pairwise distance between clades is at the high end (Dxy = 0.029, Da = 0.023) (Table 2).

The two Asian corn borer (ACB) Δ11 desaturase sequences fall into opposite clades of the ECB Δ11 desaturase genealogy. Average pairwise distance between ACB and ECB (Dxy = 0.018) is comparable to diversity within ECB (π = 0.017). The other four ECB nuclear gene genealogies [22] exhibit greater sequence divergence from the outgroup relative to π.

### Tests of neutrality

We estimated summary statistics to determine if patterns of substitution in the Δ11 desaturase genealogy deviate from equilibrium neutral expectations or from those observed at other ECB loci (Table 2). Estimates of Tajima's D [33] do not deviate significantly from the neutral expectation, although all estimates are negative, indicating a non-significant excess of low frequency polymorphisms. Fu and Li's D and F statistics [34] are significantly less than zero (P < 0.05) when we choose either of the ACB sequences as an outgroup, indicating an excess of mutations on the external branches of the tree (low frequency polymorphisms).

**Figure 4:**
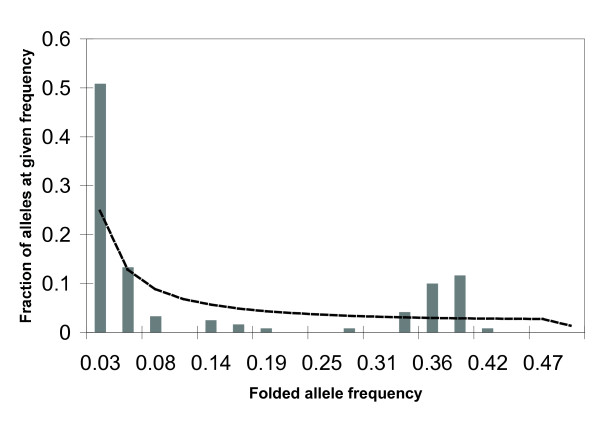
**Folded allele frequency spectrum**. The plots illustrate the expected (solid line) and observed (grey bars) frequencies of alleles, ranging from those present as singletons through those present in half (18/36) of ECB haplotypes sampled. The folded rather than unfolded spectrum is provided because the two ACB sequences are dissimilar, and therefore neither was used to infer the ancestral state. The observed allele frequency spectrum has a two-fold excess of singleton alleles (observed frequency of alleles present in a single individual = 0.508 [expected frequency = 0.248]) and three consecutive excesses of intermediate frequency alleles (observed frequency of alleles present in 12/36, 13/36, and 14/36 individuals = 0.0412, 0.100, and 0.117 [expected frequency = 0.030, 0.029, 0.028]).

We compared expected and observed allele frequency spectra (Figure 4) to determine if our sample contains an excess of intermediate frequency alleles as might be expected given the two divergent clades. This analysis confirms the excess of intermediate frequency alleles, and also displays a greater than predicted number of low frequency alleles as suggested by Fu and Li's test. The Δ11 desaturase allele frequency spectrum has a two fold excess of singletons, and three consecutive excesses of intermediate frequency alleles, one of which is three fold greater than the expected allele frequency spectrum. We performed a coalescent simulation to determine whether neutral processes in a population of varying size can account for this bimodal allele frequency spectrum. Specifically, a population bottleneck and subsequent expansion can cause estimates like Tajima's D to rapidly change in sign and intensity [35]. In our coalescent simulation, we find that the incidence of allele frequency spectra that have a two fold excess of singletons and three consecutive excesses of intermediate frequency alleles is rare: with constant population size, only one genealogy in a thousand shows this pattern and for populations that experienced a recent bottleneck/expansion, at most 31/1,000 genealogies exhibit bimodality.

## Discussion

### Variation at Δ11 desaturase does not contribute to current reproductive isolation between E and Z moths

At loci that contribute to reproductive isolation between the E and Z pheromone strains (e.g., genes involved in the specificity of pheromone production and response) we expect to find fixed differences between the E and Z strains. Absence of differentiation between E and Z pheromone strains in the Δ11 desaturase genealogy suggests that variation at Δ11 desaturase does not contribute to current reproductive isolation between E and Z moths. Failure of this locus to sort pheromone strains into exclusive groups may reflect shared ancestral polymorphism [36] and/or ongoing hybridization between the pheromone strains. In North America, it is known that hybridization does occur in natural populations [37,38], but the presence of both A and B haplotypes in ACB suggests that the polymorphism has persisted since before the divergence of ECB and ACB, which has been estimated to be 1 mya [6].

### Explanations for the existence of discrete A and B clades

Clade specific PCR demonstrates that clades A and B of our genealogy represent alleles at a single gene locus and highlights the potential difficulties in distinguishing between gene duplication and allelic variation. Several mechanisms may result in the pattern of deep divergence we observe at Δ11 desaturase, including past or present balancing selection, population substructure, or changes in population size.

The excess of intermediate frequency alleles apparent in the allele frequency spectrum (Figure 4) is a signature of balancing selection. However, Tajima's D and Fu and Li's D and F statistics do not detect this excess, perhaps because these statistics are counterbalanced by an excess of low frequency alleles or because they lack the power to detect this pattern. One potential explanation for the simultaneous excess of intermediate and low frequency alleles would be the concurrent action of balancing selection and population growth. Both a host shift to maize in Europe and the subsequent introduction of ECB into North America presumably involved brief population bottlenecks, followed by periods of population growth. Another possibility is that selection alone resulted in an excess of low and intermediate frequency alleles; for example, balancing selection could maintain two distinct alleles in the population, both of which are under purifying selection. A third possibility is that the bimodal allele frequency spectrum resulted from neutral processes (without selection) in a population of fluctuating size. However, analysis of the allele frequency spectra from data simulated under various population histories shows that a bimodal allele frequency spectrum is rare.

The persistence of discrete clade A and B alleles, in spite of direct evidence for recombination, also suggests selection. Using π as an estimate of 4Nμ, we find that the number of recombination events per mutation event in the Δ11 desaturase gene is low (γ/4Nμ = 0.459) compared with values for the PBP locus (γ/4Nμ = 1) [21] and compared to several genes in D. melanogaster [32]. Yet estimates of the minimum number of recombination events are the same for both ECB genes (Rm = 8), and the 3 sequenced recombinant Δ11 desaturase alleles show multiple recombination breakpoints. This latter evidence of recombination is not consistent with the low rate of recombination per mutation at the Δ11 desaturase locus. Instead, the apparent ineffectiveness of recombination may result from selection to maintain two distinct haplotypes. An alternate explanation for these data is that random sampling during a population bottleneck fortuitously increased the frequency of alleles at extreme ends of a polymorphic spectrum, while under-sampling from the "gradient" of haplotypes in between.

Long-term balancing selection results in gene genealogies that exhibit trans-specific polymorphism [39]. This is the pattern we observe (the outgroup ACB sequences are more closely related to ECB than to each other), but given the recent divergence and large Ne of ECB and ACB, it may be possible that ancestral polymorphisms at neutral sites persist in these sister species resulting in some loci that do not sort haplotypes along species boundaries.

In addition to balancing selection and changing population size, population substructure or recent admixture of isolated populations could also explain the two major clades observed in our genealogy. Population substructure and recent admixture seem less likely given that mtDNA and the four other nuclear gene loci for which data are available do not display any evidence of a similar deep divergence between two clades (although Tpi does show the two pheromone strains to be nearly exclusive groups [22]). Furthermore, clades A and B do not reflect any known ecological or behavioral differences (e.g., geographic region, life cycle, pheromone strain). Our clade specific PCR analysis provides additional data confirming that clades A and B do not reflect any known ecological boundaries (Additional File 5: CladeSpecificResults.xls).

### A possible scenario for balancing selection

The hypothesis that balancing selection drives differentiation at this locus, although only one of multiple possibilities, is particularly interesting because it implies a functional difference between the clade A and B Δ11 desaturase alleles that is under selection, but does not affect mating phenotype (E or Z). We find two non-synonymous fixed amino acid differences between clades A and B, but both of these differences are conservative. However, amino acid position(s) responsible for phenotypic differences between clades A and B may be in an exon that was not sequenced for this genealogy.

Lassance and Löfstedt [2] identify a dual role for the Δ11 desaturase enzyme in female and male pheromone production. They suggest the sharing of an enzymatic pathway (that includes Δ11 desaturase), but with different substrates in the two sexes. The requirement for a single enzyme to deal with multiple substrates might provide a mechanistic explanation for the maintenance of two different allelic classes in *Ostrinia* populations over considerable evolutionary time. However, in male moths, only Z strain males appear to rely on Δ11 desaturase for pheromone production [2]. In E strain ECB and in ACB, males do not use this enzyme for pheromone production. The greater abundance of Z strain moths and incomplete reproductive boundaries between the E and Z strains may account for persistence of both Δ11 desaturase allelic classes in the E strain. However, the balancing selection hypothesis is difficult to rationalize in ACB, where neither male nor female moths utilize Δ11 desaturase for pheromone production. Presence of the two allelic classes in ACB could represent ancestral polymorphism. Alternatively, the Δ11 desaturase may have an additional function, not related to pheromone biosynthesis, which could explain the maintenance of polymorphism in both ACB and ECB populations over evolutionary time.

### Evolution of pheromone mating systems

The presence of two divergent haplotypes at a locus involved in pheromone biosynthesis illustrates how genetic variation can exist in moth mating systems without directly affecting patterns of reproduction. Standing variation is a key component in hypotheses about how novel pheromone mating systems evolve in the presence of strong stabilizing selection. For example, variation may exist in the form of rare males with broader pheromone preferences than the rest of the population, which enables them to respond to novel blends [40]. In another model, variation exists in the form of silent gene duplicates for which alternate activation may lead to shifts in pheromone biosynthesis [10,13]. In a third model, variation at upstream steps of a pheromone biosynthesis pathway is masked by the action of enzymes downstream in the pathway [5]. The Δ11 desaturase gene genealogy provides a specific example of another avenue by which variation may be present in pheromone mating systems, either as a result of neutral processes and/or maintainance by selection. The dual role of the Δ11 desaturase enzyme in male and female pheromone biosynthesis provides a mechanism by which two divergent haplotypes could be maintained at this locus, and raises the question of whether other enzymes involved in pheromone production also have dual functions and harbor divergent allelic types. Most studies of enzymes involved in pheromone biosynthetic pathways in Lepidoptera have focused on patterns of variation across species or higher taxa. Our results emphasize the importance of also examining patterns of variation within populations or species.

## Conclusions

The two pheromone strains of *Ostrinia nubilalis* are not differentiated at the D11 desaturase locus, confirming that the encoded enzyme does not contribute to current reproductive isolation between the two strains. However, the genealogy is characterized by the presence of two divergent clades that differ by many fixed nucleotide substitutions and indels. Historical demography cannot easily account for this pattern, and balancing selection may be acting to maintain two distinct allelic classes at this locus. The dual role of Δ11 desaturase in both the biosynthetic pathways of male and female pheromone provides a possible functional basis for the maintenance of allelic diversity.

## Methods

### Primers

Protein and cDNA sequences for each of five expressed ECB desaturases (Δ9FB, Δ9, Δ11 (2 copies), Δ14), as well as for ACB Δ11 desaturase, were obtained from GENBANK (ECBFB-Z9: AF243047, ECBG-Z9: AF430246, ECBG-Z/E11: AF441221, ECBG-Z/E14: AF441220, ACBG-Z/E11: AF441861) [6] and aligned using Seqman gene analysis software (DNASTAR, Madison, WI). From this alignment, a primer pair was designed to bind exclusively to the Δ11 desaturase genes (Table 1). Because the two full-length ECB Δ11 desaturase copies identified by Xue et al. [25] do not differ in coding sequence, our primers amplify both. Sequence similarity searches of the remaining three ECB desaturases yielded no matches to our primer pair. Amplification with these primers, which spanned base pairs 403-781 of the cDNA sequence (Figure 1), produced a PCR product larger than 378 bp, confirming the presence of intronic sequence. This same primer pair was also used to amplify the Δ11 desaturase gene from Asian corn borer.

### Sequences

We obtained genomic DNA from a panel of 38 moths used by Dopman et al [22]. This sample includes moths from both the Z and E ECB strains in North America, from a single Z population in Europe, and from 2 ACB moths. We amplified Δ11 desaturase in PCR reactions containing 1 × Taq buffer (Invitrogen, Carlsbad, California), 1.5 mM MgCl, 200 μM each dNTP, 2 pmoles 403f, 2 pmoles 781r, 1 unit platinum Taq polymerase, and approximately 20 ng template genomic DNA in a 10 μl volume. Reactions were run for 35 cycles of 95° for 50 seconds, 50° for one minute, and 72° for one minute. Presence of insertion/deletion polymorphisms necessitated cloning of individual haplotypes to obtain reliable sequence reads. Cloning was performed using PCR 2.1-TOPO vector kits (Invitrogen) and sequencing was done in house on an ABI 377 automated sequencer with a BigDye version 3.1 cycle sequencing kit (Applied Biosystems, Foster City, California). We sequenced inserts using M13 forward and reverse primers complimentary to the PCR 2.1 TOPO vector. The large intronic regions prevented single pass sequence reads; therefore two internal sequencing primers (550f and I-267f) were developed (Table 1). Several haplotypes contained very large insertions that required development of additional primers and walk-through sequencing.

### Genealogy construction

For each individual, only one Δ11 desaturase haplotype was used in genealogy construction, and this sequence was chosen randomly by selecting only one of many clones for sequencing. DNA sequences were manipulated with the DNASTAR programs (DNASTAR, Madison, WI), maximum parsimony trees were constructed using PHYLIP [41], and maximum likelihood trees were constructed using PAUP [42] with optimum substitution models identified using ModelTest [43]. For maximum parsimony trees, gaps were coded as a "fifth base", and multiple-base indels were down-weighted to single-base indels under the assumption that these represent single evolutionary changes. Bootstrap values for the maximum parsimony genealogy were calculated in PHYLIP from 1,000 bootstrapped data sets. We also constructed maximum parsimony trees using only intron sequence or only exon sequence, and using sequence from three contiguous regions (bps 1-455, 456-910, and 911-1365).

### Testing for paralogous genes

To determine whether the two divergent Δ11 desaturase clades we observe represent separate genetic loci (gene duplicates) as predicted by Xue et. al. [25], we designed four clade specific primers at sites where there are fixed differences between the two clades (Table 1). We performed multiple PCRs on our panel of 36 ECB moths, pairing these clade-specific primers with either 403f or 781r. To increase specificity, PCRs were performed using a "touch down" technique, starting with a high annealing temperature (70°C), decreasing this temperature by one degree each cycle until 55°C, and maintaining this annealing temperature for the remaining 20 cycles. Because of the 87 bp size difference between the Δ11 desaturase variants, the specificity of each reaction could be confirmed based on the predicted size differences of the PCR products. We tested whether the two Δ11 desaturase gene classes represent paralogous gene copies as follows. If each moth possesses duplicate copies of the Δ11 desaturase locus, we expect to observe four successful PCRs for each moth (2 of the expected size for each locus). However, if these variants are actually divergent alleles at a single locus, we expect some moths (those homozygous for one variant) to produce only two successful PCRs. In this case only amplification from "heterozygous" moths (clade1/clade 2 heterozygotes) will produce four PCR products.

### Sequence analysis

We used DnaSP version 5.0 [44] to calculate average nucleotide diversity (π in [29]), the average number and average net number of nucleotide differences between populations (D_xy _and D_a _in [29]), the minimum number of recombination events (R_m _in [30]), the per gene and per site recombination parameters R = 4Nr and C = 4Nc [31], Tajima's D [33], Fu and Li's D and F statistics [34] with ACB as an outgroup, and the expected and observed allele frequency spectra [33]. Indel polymorphism was excluded from analyses using DnaSP. We also used SITES [32] to compute the per site recombination parameter γ.

### Coalescent simulation

We used msθ [45] to generate coalescent genealogies with the same number of samples, segregating sites, and recombination events as we find in the Δ11 desaturase sample. These genealogies were generated assuming either a constant population size, or a recent population bottleneck followed by exponential growth to the current population size N_0_. We varied the time since the bottleneck (T_1_) from 0.001 to 0.025 in steps of 0.01, and the length of the bottleneck from 0.0 (instantaneous) to 0.499 in steps of 0.025 (all units are in terms of 4N_0 _generations). We also varied the size of the bottlenecked population (N_B_) as a fraction of the current population (N_0_) by allowing the growth rate after the bottleneck (α in the equation N_B _= N_0_e^-αT1^) to range from 1 to 296 in units of 5. The resulting values for N_B _ranged from ~0 (strong bottleneck) to ~1 (no bottleneck). For each variation of population demography, we generated 1,000 genealogies and asked for how many of these 1,000 genealogies does the allele frequency spectrum resemble that of Δ11 desaturase. To satisfy this condition, we searched for allele frequency spectra that have at least a two-fold excess of singletons, and at least three consecutive excesses of intermediate frequency alleles (defined as alleles with frequency 0.138 - 0.5) when compared to the folded expected allele frequency spectrum. For intermediate frequency alleles, we define an excess as when the simulated allele frequency spectrum is greater than the expected.

## Authors' contributions

KG carried out the molecular genetic studies, sequence alignment, genealogy construction, and data analyses, participated in study design, and drafted the manuscript. RH conceived of the study and the study design, participated in each step of data analysis and interpretation, and helped to draft the manuscript. All authors read and approved the final manuscript.

## Supplementary Material

Additional file 1 **Position and length of insertions larger than 45 bp**. Several Δ11 desaturase alleles have unique large insertions in intron 1 or 2. This table annotates the position and length of those insertions larger than 45 bp. Those alleles labeled with a * have insertions at the same site that differ in nucleotide composition. Click here for file

Additional file 2 **Genealogies based only on (a) exon sequences and (b) intron sequences**. Shown are the maximum parsimony gene genealogies with outgroup sequences labeled in bold. See Figure 2 for further details. Click here for file

Additional file 3 **Regional gene genealogies for (a) 1-455 bp (b) 456-910 bp (c) 911-1365 bp**. Shown are the maximum parsimony gene genealogies with outgroup sequences labeled in bold. See Figure 2 for further details. Click here for file

Additional file 4 **Maximum likelihood genealogy**. Shown is the maximum likelihood gene genealogy constructed using the HKY85 + G model with outgroup sequences labeled in bold. See Figure 2 for further details. Click here for file

Additional file 5 **Results of clade specific PCRs**. Clade specific primers were used to determine whether each ECB moth possesses a gene copy from both clades A and B in a pattern consistent with fixed gene duplication. This table displays the results of these clade specific PCRs. A number in parentheses indicates successful amplification with that primer pair and the length of the amplified PCR product. The clade specific forward and reverse primers differ in sequence, but are at the same nucleotide position. Despite identical primer positioning, six fixed indel polymorphisms between clades result in an 87 bp difference in expected PCR product size. These size differences allow us to confirm amplification of an allele from one clade versus another. We indicate where the PCR product is significantly (>100 bp) larger than predicted. In all of these cases, the original PCR with primers 403-781 confirms that these individuals have larger amplification products, perhaps due to additional unique insertions. Also indicated is the identity of the allele sequenced for the genealogy. A^ $ ^symbol indicates that the sequenced genotype was a recombinant. The final column shows the predicted genotype of each ECB moth, based on the combination of sequencing and clade-specific PCR. Click here for file
