# Supplementary material for: A Δ11 desaturase gene genealogy reveals two divergent allelic classes within the European corn borer (Ostrinia nubilalis)
Source: BMC Evol Biol. 2010 Apr 27;10:112. doi: 10.1186/1471-2148-10-112 (PMC2877688; doi:10.1186/1471-2148-10-112)
Supplement: Additional file 1 — Position and length of insertions larger than 45 bp. Several Δ11 desaturase alleles have unique large insertions in intron 1 or 2. This table annotates the position and length of those insertions larger than 45 bp. Those alleles labeled with a * have insertions at the same site that differ in nucleotide composition. [file 1471-2148-10-112-S1.PDF]

Sequence	Insertion Site	Insertion Length
NYZ16<br>HUNZ14<br>ACB2	Intron 1:<br>Position 35 of 1365bp	112bp
NYE32	Intron 2:<br>Position 1,036 of 1365bp	48bp
NYE28	Intron2:<br>Position 1,096 of 1365bp	776bp
NYE32*<br><br>NYZ20*	Intron 2:<br><br>Position 1,152 of 1365bp	NYE32: 284bp<br>NYZ20: 922bp
