# Supplementary figures and images for: A Δ11 desaturase gene genealogy reveals two divergent allelic classes within the European corn borer (Ostrinia nubilalis)
Source: BMC Evol Biol. 2010 Apr 27;10:112. doi: 10.1186/1471-2148-10-112 (PMC2877688; doi:10.1186/1471-2148-10-112)

**(a)**

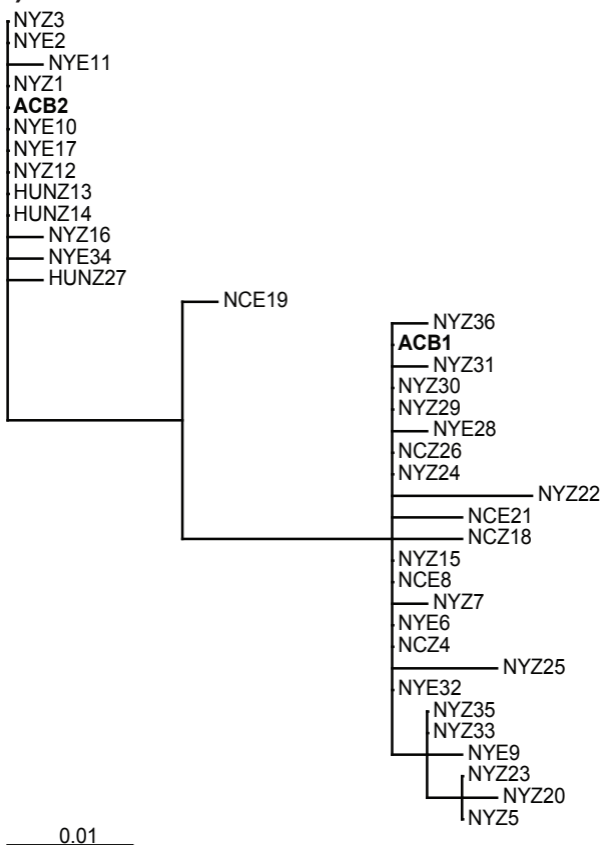

**(b)**

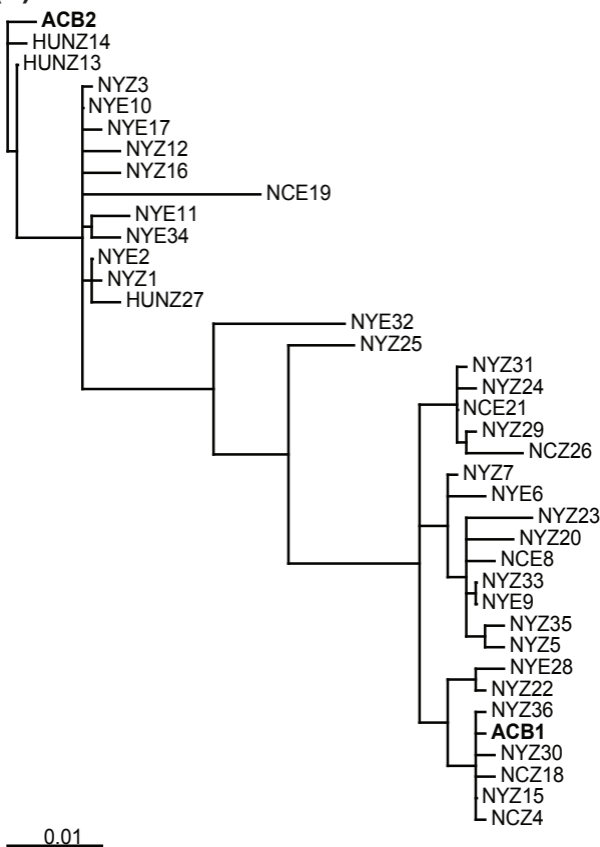

Supplement: Additional file 2 — Genealogies based only on (a) exon sequences and (b) intron sequences. Shown are the maximum parsimony gene genealogies with outgroup sequences labeled in bold. See Figure 2 for further details. [file 1471-2148-10-112-S2.PDF]

(a)

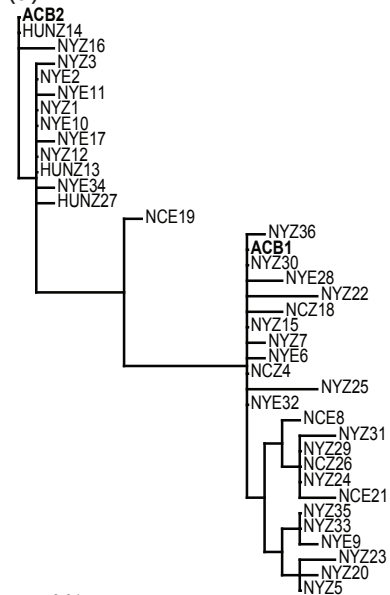

(b)

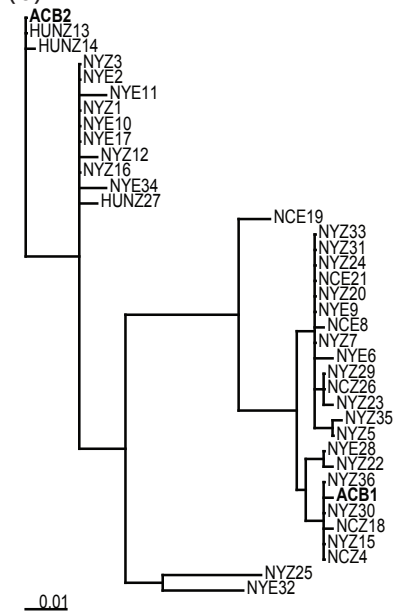

(c)

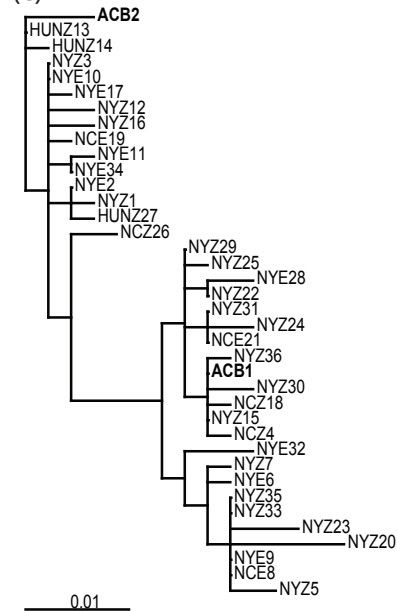

Supplement: Additional file 3 — Regional gene genealogies for (a) 1-455 bp (b) 456-910 bp (c) 911-1365 bp. Shown are the maximum parsimony gene genealogies with outgroup sequences labeled in bold. See Figure 2 for further details. [file 1471-2148-10-112-S3.PDF]

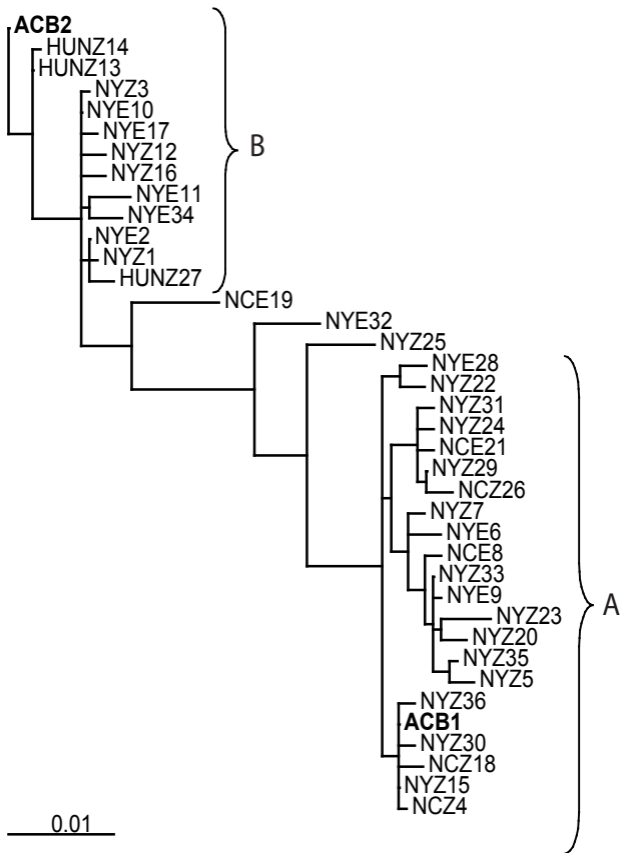

Supplement: Additional file 4 — Maximum likelihood genealogy. Shown is the maximum likelihood gene genealogy constructed using the HKY85 + G model with outgroup sequences labeled in bold. See Figure 2 for further details. [file 1471-2148-10-112-S4.PDF]
